# Ratings of valence, arousal, happiness, anger, fear, sadness, disgust, and surprise for 24,000 Dutch words

**DOI:** 10.3758/s13428-023-02239-6

**Published:** 2023-10-02

**Authors:** Laura J. Speed, Marc Brysbaert

**Affiliations:** 1 Centre for Language Studies, Radboud University, Nijmegen, Netherlands; 2 Department of Experimental Psychology, Ghent University, Ghent, Belgium

**Keywords:** Valence, Arousal, Discrete emotions, Lexical decision, Personality

## Abstract

Emotion is a fundamental aspect of human life and therefore is critically encoded in language. To facilitate research into the encoding of emotion in language and how emotion associations affect language processing, we present a new set of emotion norms for over 24,000 Dutch words. The emotion norms include ratings of two key dimensions of emotion: valence and arousal, as well as ratings on discrete emotion categories: happiness, anger, fear, sadness, disgust, and surprise. We show that emotional information can predict word processing, such that responses to positive words are facilitated in contrast to neutral and negative words. We also demonstrate how the ratings of emotion are related to personality characteristics. The data are available via the Open Science Framework (https://osf.io/9htuv/) and serve as a valuable resource for research into emotion as well as in applied settings such as healthcare and digital communication.

## Introduction

Emotions are an important aspect of life. Therefore, human language contains many emotion-related words. Yet psychology researchers have been slow to study the impact of emotional connotation on word processing, perhaps due to the computer metaphor that dominated the cognitive revolution (Eysenck & Brysbaert, 2023). This emphasized the importance of form-related aspects of words, such as word length, similarity to other words, frequency of encounter, and order of acquisition. A widely studied effect, for example, was the finding that high-frequency words are processed more efficiently (faster, and with fewer errors) than low-frequency words (Balota & Chumbley, 1985; Brysbaert et al., 2018).

Research on the influence of emotions on word processing was initiated by authors working on attention and emotion. In this work, emotion was considered in terms of a positive – negative valence dimension. For example, it was observed that participants (especially patients) responded more slowly to negative words than to positive and neutral words in a Stroop task (for a review, see Williams et al., 1996). This research was criticized by Larsen et al. (2008), who pointed out that the negative words used in studies of emotional Stroop were often longer and less frequent than the neutral or positive control words. When Larsen et al. (2008) controlled for these confounds, they no longer found that negative words elicited slower responses than neutral words.

In order to more precisely study the effect of emotion on word processing, individual ratings of valence have been collected (e.g., Bradley & Lang, 1999; Moors et al., 2013; Warriner et al., 2013). Subsequent studies with much larger stimulus sets (Kousta et al., 2009; Kuperman et al., 2014; Ponari et al., 2018; Yap & Seow, 2014) established that word valence does affect word processing times. Initially, an inverted-U effect of valence on response time was reported for English, with faster responses to negative and positive words compared to neutral (Kousta et al., 2009; Kuperman et al., 2014; Vinson et al., 2014; Yap & Seow, 2014). However, when the interaction between valence and word frequency was taken into account, a linear effect of valence was revealed: positive words are processed faster than neutral words and negative words (Kuperman et al., 2014). This pattern was confirmed in a recent large-scale analysis by Gao et al. (2022). They reported that in all English lexical decision megastudies analyzed, positive words were responded to faster than neutral words and negative words. However, an intriguing difference for negative words was found between visual and auditory lexical decision. Written negative words were responded to as slowly as neutral words, but in auditory lexical decisions they elicited faster responses: Negative spoken words were responded to as quickly as positive spoken words, and both elicited faster responses than neutral words. The authors interpreted the fast responses to negative words in auditory lexical decision as the outcome of a bias to possible danger in audition.

Emotion related to words is typically estimated with valence and arousal ratings (Warriner et al., 2013), dimensions thought to be orthogonal. Valence refers to the degree to which a stimulus evokes negative or positive feelings; arousal indicates the degree to which a stimulus is calming or arousing. The dimensions go back to seminal work by Osgood et al. (1957) who used the terms *evaluation* (valence) and *activity* (arousal). Kuperman et al. (2014) reported that both valence and arousal explain variance in word processing times, but that the effect of arousal (arousing words are recognized more slowly than calming words) is much smaller than that of valence. The work of Osgood et al. (1957) distinguished a third dimension, potency (referring to the degree of control exerted by the stimulus), but this dimension is strongly correlated with valence (Warriner et al., 2013), so it is usually disregarded in word recognition studies.

**Fig. 1:**
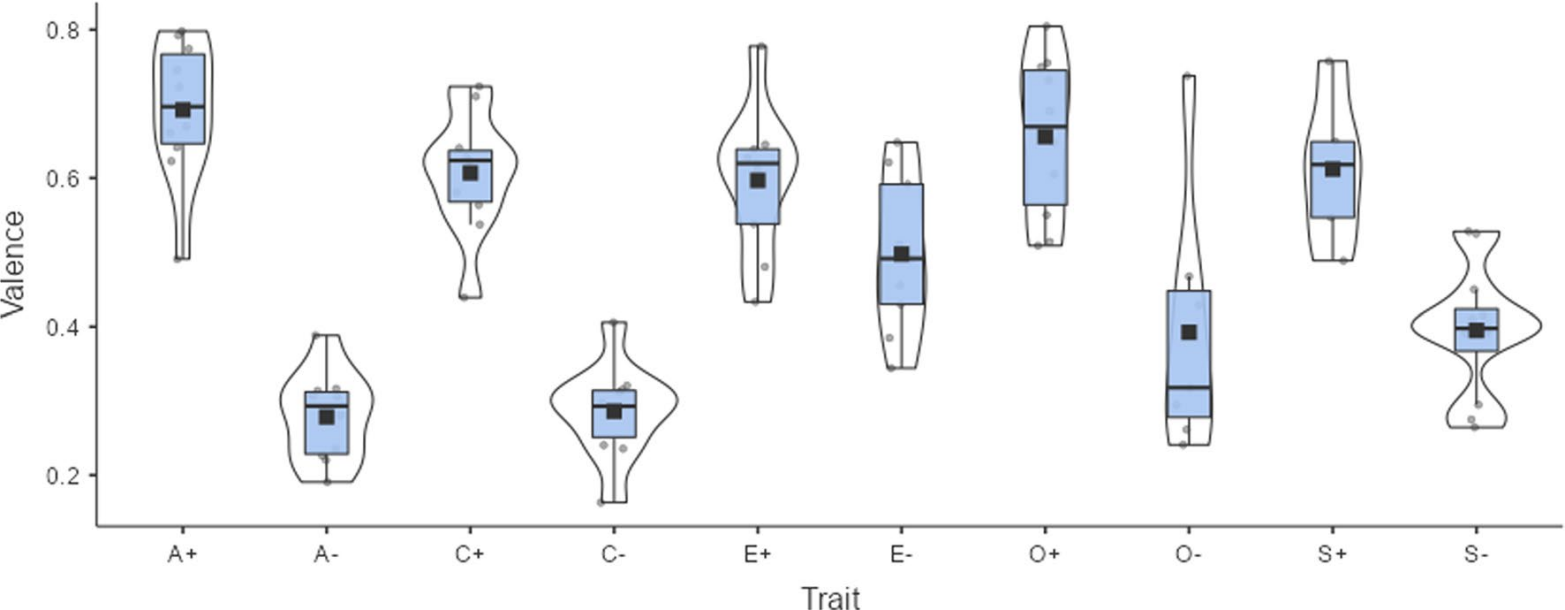
Valence of Big Five personality traits, based on adjectives reported by Goldberg (1992) and estimates obtained by Hollis et al. (2017). 0 = very negative, 1 = very positive. A+/A- = positive/negative pole of agreeableness, C+/C- = positive/negative pole of conscientiousness, E+/E- = positive/negative pole of extraversion, O+/O- = positive/negative pole of openness, S+/S- = positive/negative pole of emotional stability. Figure created with The jamovi project (2022)

**Fig. 2:**
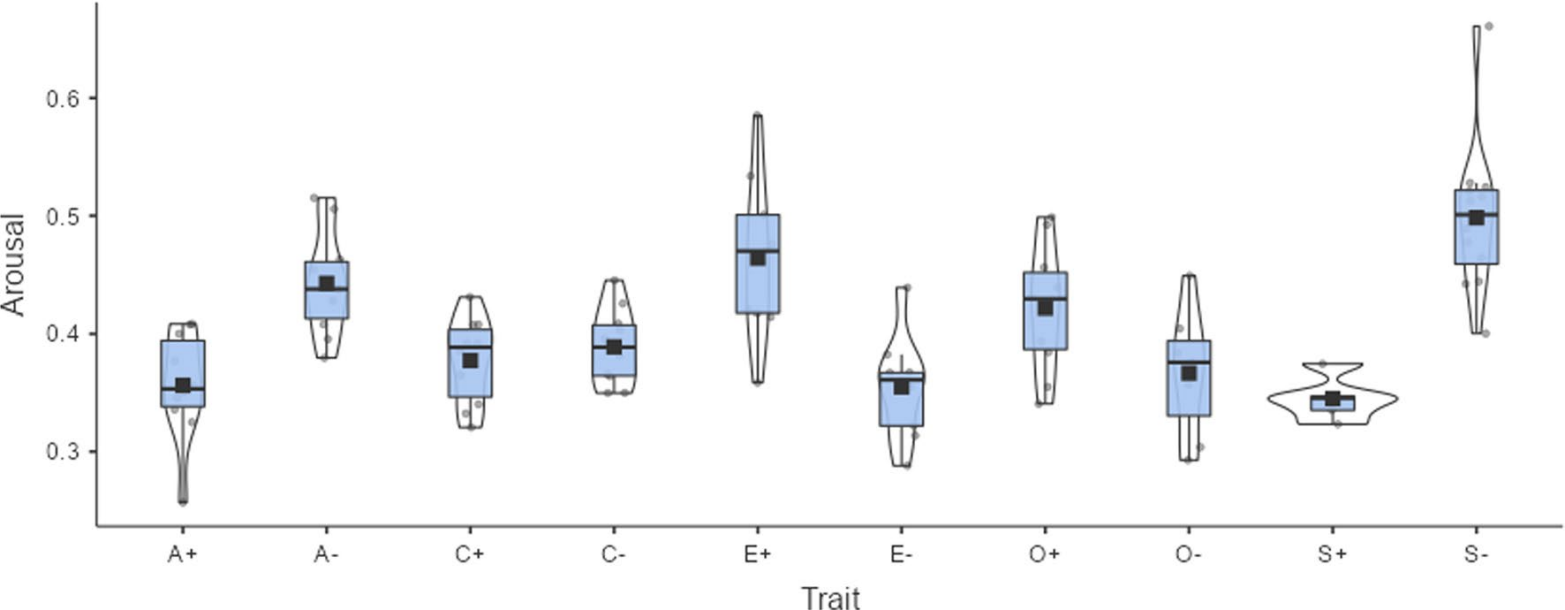
Arousal evoked by Big Five personality traits, based on adjectives reported by Goldberg (1992) and estimates obtained by Hollis et al. (2017). 0 = no arousal at all, 1 = high arousal. A+/A- = positive/negative pole of agreeableness, C+/C- = positive/negative pole conscientiousness, E+/E- = positive/negative pole extraversion, O+/O- = positive/negative pole of openness, S+/S- = positive/negative pole emotional stability

Beyond differences in word processing, valence and arousal ratings can also be used to estimate attitudes expressed by words. For example, ratings of thousands of adjectives used to describe people's personalities have been analyzed. Factor analysis indicates that they group into five bipolar traits: (1) agreeableness versus hostility, (2) conscientiousness versus carelessness, (3) emotional stability versus neuroticism, (4) extraversion versus introversion, and (5) openness to experience versus narrow-mindedness. Valence and arousal ratings can be used to describe these five personality factors. Ninety-two of the 100 adjectives characterizing the poles of the Big Five traits published by Goldberg (1992) are available in the emotion norms published by Hollis et al. (2017), estimated based on Warriner et al. (2013). These norms provide values for 75,000 English words based on semantic vectors and extrapolation from human ratings. Figure 1 shows the valence associated with each trait; Fig. 2 shows the degree of arousal evoked by the traits. Both figures were created with jamovi (The jamovi project, 2022).

All traits have higher valence at their positive pole. People positively rate persons who are agreeable, conscientious, extroverted, open, and emotionally stable. They negatively rate unagreeable and unconscientious persons. The largest difference is observed for agreeableness versus hostility and the smallest for extraversion versus introversion. As for arousal ratings, agreeable and emotionally stable people are less arousing than their opposites; the same is true to a lesser extent for narrow-minded people. This analysis demonstrates the potential of emotion ratings to investigate the perceived emotion associated with specific semantic or social categories.

One limitation of the valence/arousal coding scheme is that it provides little information about the underlying emotions. For example, the observation that negative words are recognized more quickly than neutral words in spoken form but not in written form says little about the underlying emotions. Gao et al. (2022) speculated that negative spoken words attract attention because they signal danger (as suggested by Murphy et al., 2013), in line with an adaptive explanation of a broader negativity bias in humans (Rozin & Royzman, 2001). If this is true, then faster reaction times should be limited to fear-inducing words and should not be observed with sadness-inducing words. Moreover, knowing that hostile people elicit negative reactions does not tell us whether these reactions are related to anger, fear, sadness, or disgust.

An alternative to dimensional models of valence and arousal are discrete emotion categories. In such models, a distinction is usually made between six emotions that can be expressed and recognized via facial expressions and thought to have their own neurological response patterns (Revers et al., 2022): happiness, anger, fear, sadness, disgust, and surprise (Briesemeister et al., 2011; Ric et al., 2013; Riegel et al., 2016; Stadthagen-González et al., 2018; Stevenson et al., 2007; Syssau et al., 2021).

**Fig. 3:**
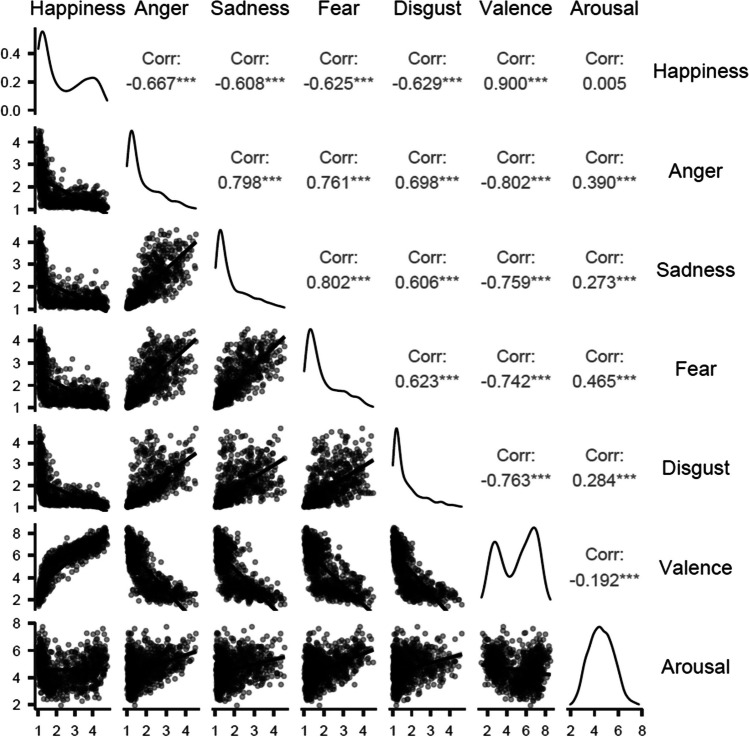
Correlations between discrete emotions and valence/arousal for 1027 English words. Analyses conducted with jamovi (The jamovi project, 2022)

Stevenson et al. (2007) collected ratings of five English discrete emotion ratings. Figure 3 shows the correlations between these five emotions and the valence and arousal ratings collected by Warriner et al. (2013) for 1027 shared words. It shows that valence correlates positively with happiness, and negatively with anger, sadness, fear, and disgust. Arousal correlates positively for all emotions except happiness, and correlates mostly with fear and anger. Overall, the strength of correlation varies, suggesting using only valence and arousal may misestimate the role of emotion in language.

Emotion is clearly a fundamental aspect of human psychology and the language we use. In order to build on the existing work examining the relation between language and emotion, we provide a new set of emotion ratings of Dutch words. In the remainder of this article, we discuss emotion ratings we collected for 24,000 Dutch words, across a broad class of words. This represents the largest collection of emotion ratings for Dutch words to-date. Words were sampled from a range of categories and not for their emotionality, thereby reflecting natural language. In addition to valence and arousal, we collected ratings for six primary emotions that can be communicated via facial expression: happiness, anger, fear, sadness, disgust, and surprise. Inclusion of surprise was interesting because it is generally a positive emotion, making the ratings less centered on negative feelings. These ratings will allow us to go deeper into the issues mentioned above, regarding how effects of emotional valence and arousal may differ depending on discrete emotion categories.

To validate the new set of ratings, we calculate their reliability and compare the ratings with existing emotion ratings for Dutch (Moors et al., 2013). We then explore how emotion is distributed throughout our large word set and how the emotional variables are related to each other and other relevant lexical information. To further test the validity of our ratings, we use them to predict performance in a lexical decision task. Finally, to demonstrate how the emotion ratings could be used in research beyond word processing, we use the new set of ratings to investigate how valence and arousal are related to the Big Five personality traits. All materials, ratings, and analyses are openly available at https://osf.io/9htuv/.

## Method

### Materials

The materials consisted of 24,036 words that were rated on word concreteness and age of acquisition (AoA) in Brysbaert et al. (2014), and that were known to 90% of the raters at that time. The stimuli were also used for the sensory modality norms collected by Speed and Brysbaert (2022).

For the valence and arousal ratings, the words were randomly divided over five lists of 4800 words each. For the discrete emotion ratings, words were randomly divided over 24 lists with 1000 words each.

### Participants

Participants were students from Ghent University (18–26 years old; two-thirds were female). They were members of the participant pool or they contacted us after word of mouth. Each participant completed a list of words that took them on average 3.5 h, for which they received €40. A different permutation was used for each participant to minimize sequence effects. Participants with ratings that correlated more than .10 with those of the rest were given the opportunity to complete up to five lists under the same conditions. A new list was given when the previous one was returned and validated. Nearly all returned lists were valid, although several participants did not return their list (arguably because they stopped after a few lines). Participants had to be native speakers of Dutch.

For all ratings, about two-thirds of the participants were female, in line with the typical bias in psychology studies.^1^ We started with a minimum number of eight participants per list. Extra participants were added if the reliability of a list was lower than .80 (see below for one exception). The number of participants that rated each word on a dimension ranged from 8 to 30. Columns M–N in the data file indicate the number of participants that rated each word on each dimension.

### Procedure

Participants could complete the list at their own pace at home. They were asked to find a quiet place and told they could complete the list in as many sessions as suited them.

For the valence ratings, they received an Excel file with 4800 words and were asked to judge how they felt while reading each word. The scale ranged from 1 (very negative/unpleasant) to 5 (very positive/pleasant). For the arousal ratings, the same lists were used, and participants were asked to judge from 1 to 5 how calm/passive or excited/active they felt upon reading the word.

For the discrete emotion ratings, participants received an Excel file with 1000 words and were asked to indicate how strongly they experienced each of the following emotions upon reading the word: happiness, anger, fear, sadness, disgust, and surprise. Likert scales with five alternatives were used, going from 1 (not) to 5 (very much). Each emotion was presented in a different column, with the column name always clearly visible. The order of columns (happiness, anger, fear, sadness, disgust, surprise) was the same for all lists.

For all ratings, participants could indicate if they did not know the word well enough to provide a rating. This column was added to make sure that no ratings would be given for words unknown to the rater. We also alerted the raters to the fact that it was possible that they would have to use a lot of small ratings because only a subset of the words in a language involves emotions. We told them that the use of low ratings was not a problem, as long as they took the task seriously. We felt that otherwise, raters might be inclined to give higher numbers than they felt.

## Results

Individual ratings were removed when they were outside of the scale (i.e., typos: 23 for valence, 11 for arousal, and 5 for primary emotions). For each scale, participants’ ratings were compared with the mean ratings across all participants that completed the same list, and they were removed if the correlation was less than .1 on any scale (one participant for arousal, three for valence, and three for primary emotions). Items were removed if they were rated as unknown by more than 50% of participants in a list (48 in total) and one item was removed because it appeared twice in the same list. The ratings of several participants who had incorrectly completed the primary emotions questionnaire using a 0–5 scale were transformed to a 1–5 scale.

### Descriptive statistics

Data were analyzed in R (R Core Team, 2020). Table 1 gives the main descriptive statistics of the different variables. This shows that we achieved the goal of .8 reliability for all variables, except for surprise. It is not clear to what extent this is due to individual differences in opinion about this feature, or to the fact that it was the last variable to be rated per word. Emotion norms collected in Polish found large variability in ratings of valence for words related to surprise, suggesting some people perceive surprise as positive and other perceive it as negative (Wierzba et al., 2021).

**Table 1:** Summary statistics per dimension. Mean, standard deviation, intraclass correlation, and the average number of raters per list (total number of raters/number of lists)

Dimension	*M*	*SD*	*ICC2k*	*N*_raters_
Valence	2.91	0.64	0.87	18.8 (94/5)
Arousal	3.03	0.46	0.81	15.6 (78/5)
Happiness	1.59	0.69	0.82	9.8 (235/24)
Anger	1.39	0.60	0.86	9.8 (235/24)
Fear	1.44	0.60	0.81	9.8 (235/24)
Sadness	1.38	0.57	0.83	9.8 (235/24)
Disgust	1.31	0.48	0.79	9.8 (235/24)
Surprise	1.37	0.39	0.57	9.8 (235/24)

To further assess the reliability of the valence and arousal ratings, we compared the new norms with the existing norms of Moors et al. (2013). There were 4044 words in common in the two datasets. There was a strong correlation between the two datasets for valence, *r* = .97 *p* < .001, and arousal, *r* = .84, *p* < .001.

The words *geliefde* (lover) and *vrolijk* (cheerful) had the highest valence score (4.85) and *slachtpartij* (massacre) and *verkrachter* (rapist) had the lowest (1). The word *moordaanslag* (assassination attempt) had the highest arousal score (4.71) and *passief* (passive) had the lowest (1.25). In terms of primary emotions, the words *lachen* (laugh) and *gelukkig* (happy) had the highest score for happiness (5), *kwaadheid* (anger) had the highest score for anger (4.91), *atoomwapen* (nuclear weapon) had the highest scores for fear (5), *overlijden* (death) and *droevig* (sorrow) had the highest score for sadness (5), *necrofiel* (necrophile) and *walging* (disgust) had the highest scores for disgust, and *verrassend* (surprising) had the highest score for surprise (5). Many words were not associated with any of the primary emotions; i.e., they had an average rating of 1 (proportion happiness: .20, anger: .33, fear: .26, sadness: .31, disgust: .35, surprise: .16).

### Distribution of scores

**Fig. 4:**
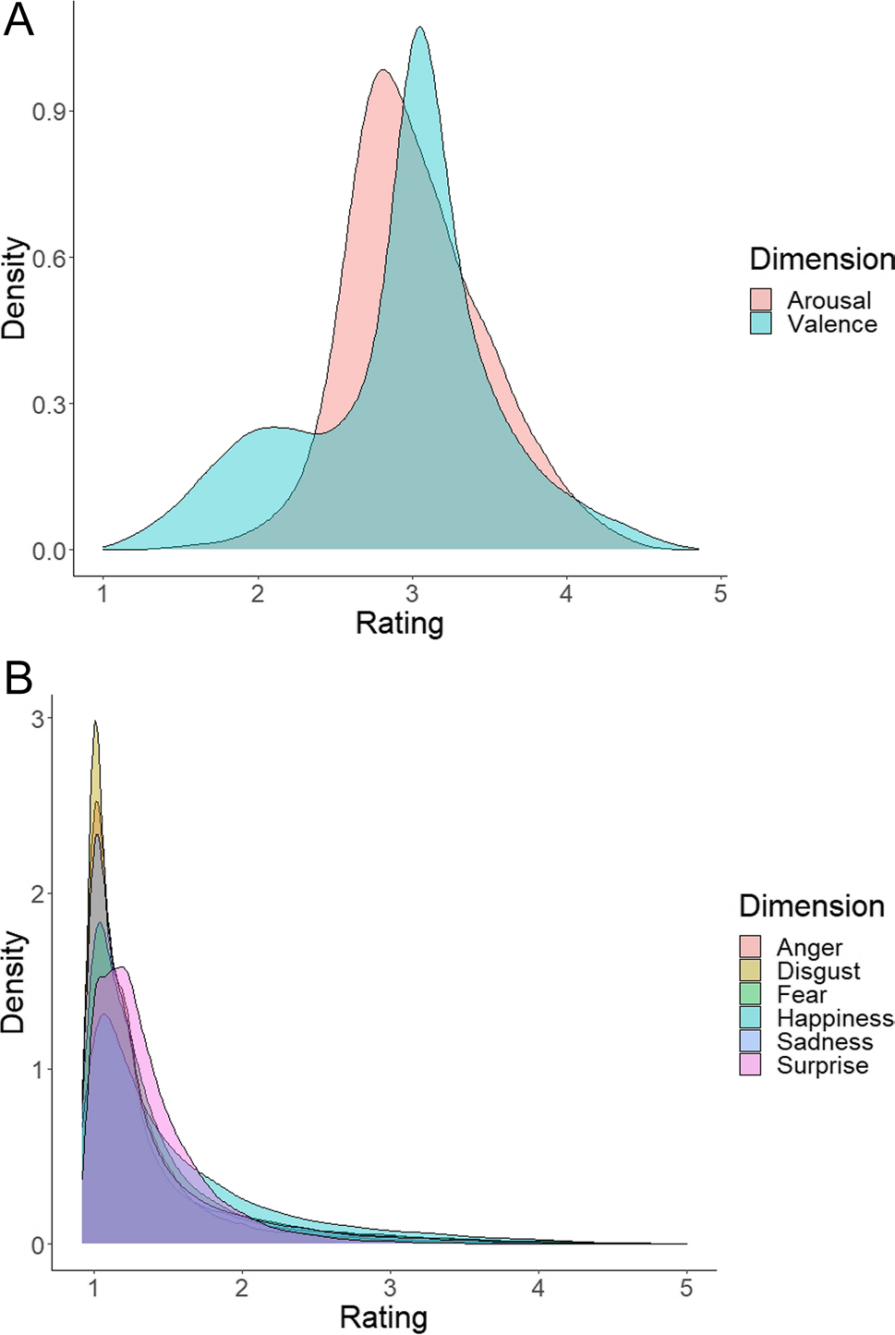
Distribution of **A** valence and arousal, and **B** primary emotions

Figure 4, created with ggplot2 in R (Wickham et al., 2016), shows the distribution of valence and arousal ratings (A) and the distribution of discrete emotion ratings (B). Whereas the distributions of valence and arousal are roughly symmetrical, those of the primary emotions are very skewed, with lots of low ratings. In the valence ratings, there is a separate bump of negative words. When interpreting the distributions, it is important to keep in mind that the distributions represent the vast majority of known Dutch words that are not transparent compounds (which are also written as a single word in Dutch). All previous norming studies had a preponderance of words selected for emotion research (see Warriner et al., 2013, Fig. 1, for data that come closest to the valence and arousal ratings of our study).

### Standard deviation of the norms

**Fig. 5:**
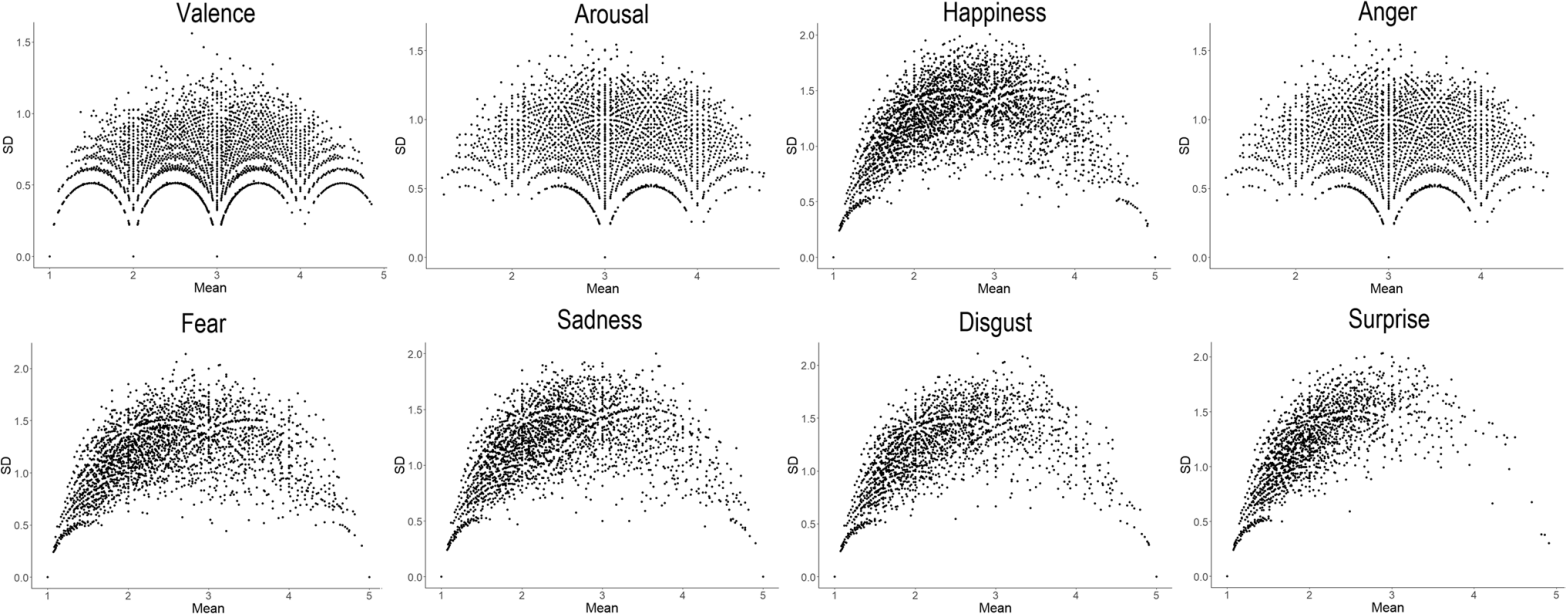
Standard deviations plotted against mean ratings across all dimensions

For all variables, standard deviations are highest in the middle range, as can be expected from range restriction at the extremes of the scale (Fig. 5). However, in particular for valence and arousal, throughout the range there are words with low standard deviations. The standard deviation of ratings is important because it can easily be confounded with average value of the stimuli selected (Pollock, 2018) and because it has been found that words with divergence in ratings are better remembered in memory studies (Brainerd et al., 2022a, b).

### Correlations between variables

Correlation analyses were conducted between all variables as well as a number of lexical variables (Table 2). Valence ratings were negatively correlated with ratings of arousal, anger, fear, sadness, and disgust, and positively correlated with ratings of happiness and surprise, although the latter correlation was close to zero. Arousal ratings were significantly negatively correlated with ratings of happiness, and significantly positively correlated with ratings of anger, fear, sadness, disgust, and surprise. Anger, fear, sadness, disgust, and surprise were all positively correlated with each other. Happiness was positively correlated with surprise but negatively correlated with all other variables.

**Table 2:** Zero-order correlations between emotion variables and lexical variables

	Arousal	Valence	Happiness	Anger	Fear	Sadness	Disgust	Surprise	Freq.	Prev.	Length	Nsyl	N_phon.	OLD20
Arousal	1	– .32**	– .13**	.47**	.45**	.25**	.30**	.31**	– .02**	0.01	.10**	.10**	.07**	– .02**
Valence		1	.70**	– .67**	– .60**	– .65**	– .58**	.02**	.01*	.10**	– .02**	– .001	– 0.01	.02**
Happiness			1	– .31**	– .28**	– .29**	– .28**	.30**	.02**	.13**	– .02**	– .02**	– 0.01	.02**
Anger				1	.60**	.65**	.60**	.19**	– .01	– 0.01	.06**	.05**	.05**	– .01
Fear					1	.64**	.46**	.33**	– 0.01	.05**	.08**	.06**	.07**	.04**
Sadness						1	.48**	.18**	– 0.004	.01*	.07**	.06**	.06**	0.00
Disgust							1	.09**	– .01*	– .04**	.01	– .01	.003	– .002
Surprise								1	– .010	.09**	.06**	.05**	.06**	.02**
Frequency									1	.04**	– .09**	– .07**	– .09**	– .07**
Prevalence										1	– .04**	– .12**	– .06**	– .12**
Length											1	.80**	.93**	.74**
Nsyl												1	.83**	.59**
N_phonemes													1	.76**
OLD20														1

In terms of lexical variables, word prevalence was positively correlated with valence, happiness, fear, sadness, and surprise, but negatively correlated with disgust. Word frequency was positively correlated with valence and happiness, but negatively correlated with arousal and disgust. Length was positively correlated with arousal, anger, fear, sadness, and surprise, but negatively correlated with valence and happiness. Number of syllables (Nsyl) was positively correlated with arousal, anger, fear, sadness, and surprise, but negatively correlated with happiness. Number of phonemes (N_phonemes) was positively correlated with arousal, anger, fear, sadness, and surprise. OLD20 (similarity to other words) was positively correlated with valence, happiness, fear, and surprise.

In summary, positive words tend to be known by more people (prevalence), occur more often in the language (frequency), and are predominantly short. This is in line with the positivity bias previously reported in language research (Dodds et al., 2015).

**Fig. 6:**
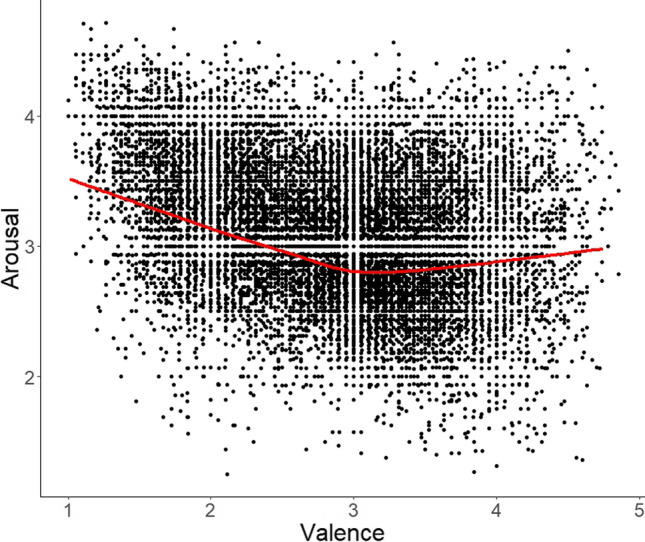
Scatterplot of arousal vs. valence with lowess line in *red*

**Fig. 7:**
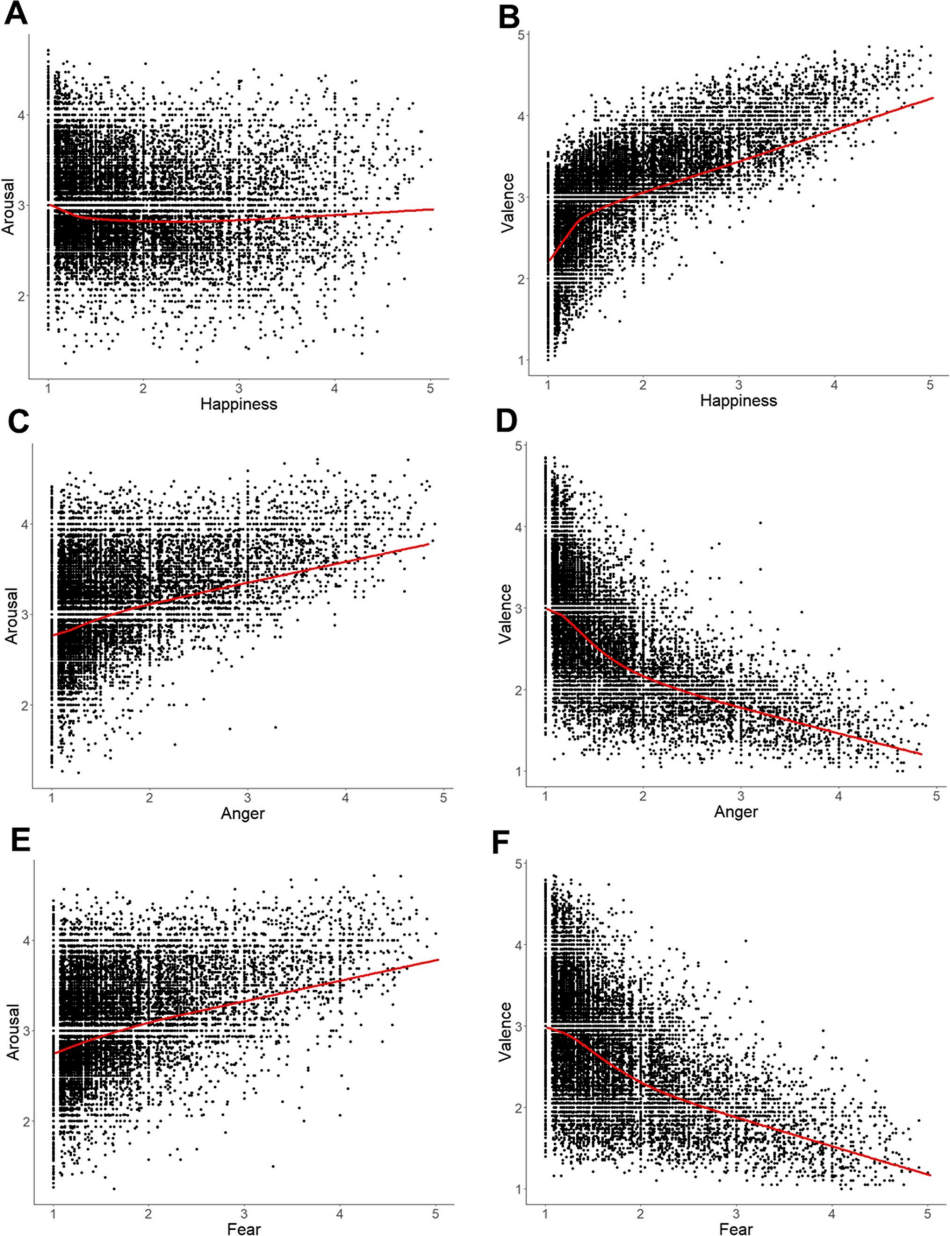

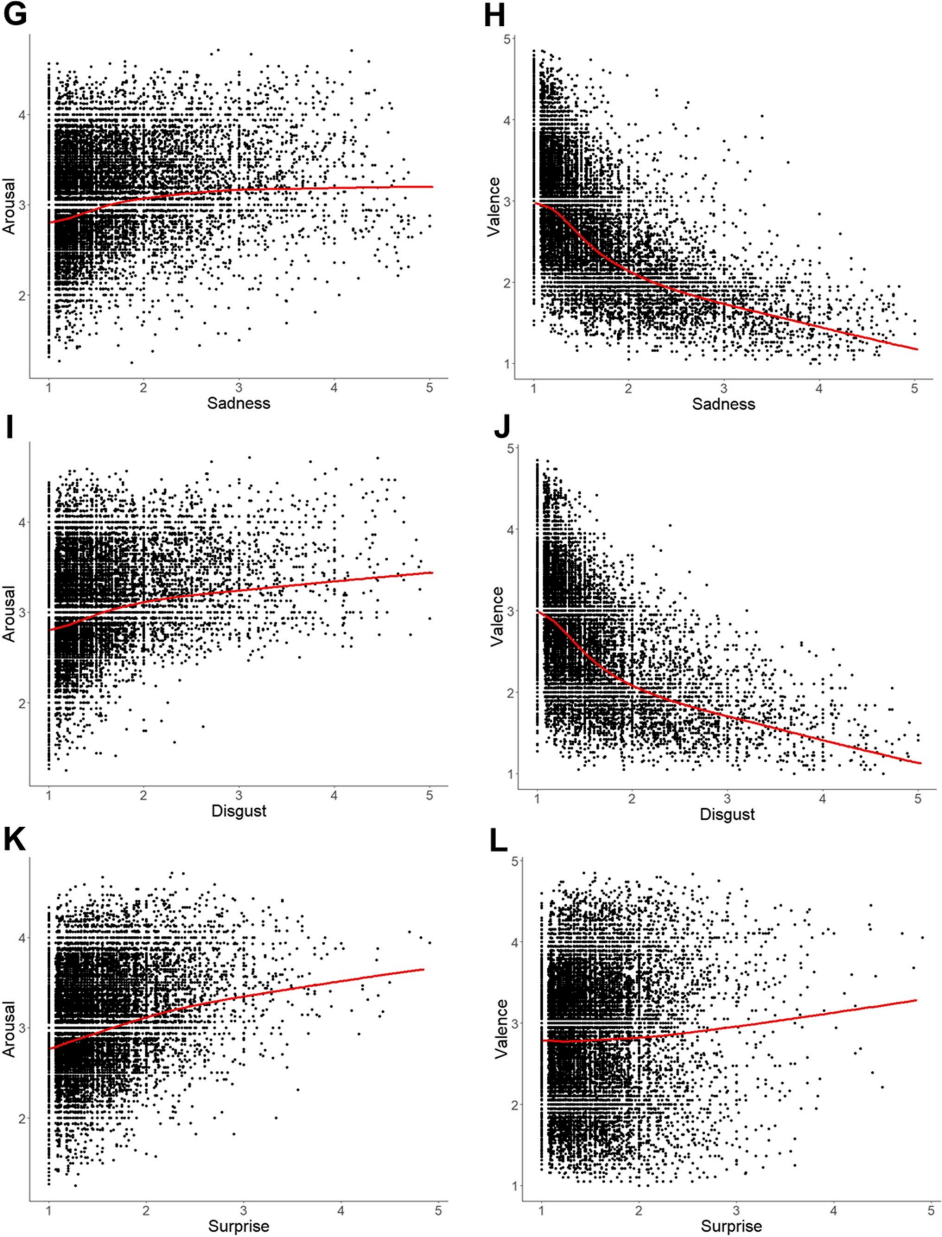
Scatterplots of all primary emotion dimensions vs. arousal and valence with lowess lines in red: (**A**) happiness vs. arousal, (**B**) happiness vs. valence, (**C**) anger vs. arousal, (**D**) anger vs. valence, (**E**) fear vs. arousal, (**F**) fear vs. valence

Because correlations only represent the linear relationship between variables, Figs. 6 and 7 give the main scatterplots. Although there are some deviations from a linear relationship, there is no evidence for a clear curvilinear curve. So, the correlations in Table 2 represent the main connections between the variables.

### Primary emotion categories

Words were assigned to a primary emotion based on their highest emotion rating (2593 words did not have one dominant primary emotion). The largest proportion of words were words dominant in happiness, accounting for 43% of all words. This supports a Pollyanna effect in language (Dodds et al., 2015; Garcia et al., 2012). Table 3 displays the distribution of ratings across emotion dimensions and discrete categories.

**Table 3:** Distribution of words over the six primary emotions with mean ratings. *Rows* are ratings in each dimension, and the *columns* are the dominant emotion category

Dimension	Dominant emotion					
	Anger	Disgust	Fear	Happiness	Sadness	Surprise
Arousal	3.38	3.04	3.28	2.90	2.96	3.07
Valence	2.22	2.52	2.47	3.37	2.31	3.01
Happiness	1.13	1.16	1.17	2.10	1.15	1.38
Anger	2.50	1.41	1.57	1.10	1.59	1.18
Fear	1.67	1.35	2.34	1.15	1.65	1.30
Sadness	1.72	1.36	1.58	1.12	2.37	1.18
Disgust	1.61	2.14	1.41	1.10	1.36	1.13
Surprise	1.37	1.21	1.45	1.34	1.30	1.83
*N*	2408	1772	3010	10273	2081	1849

### Valence and lexical decision

Regression analyses were conducted in R (R Core Team, 2020) on raw lexical decision response times taken from the Dutch Lexicon Project 2 (DLP2; Brysbaert et al., 2016) and the Dutch Crowdsourcing study (DCP; Brysbaert et al., 2019). We conducted hierarchical regression to assess the unique variance in lexical decision explained by the emotional variables.

**Fig. 8:**
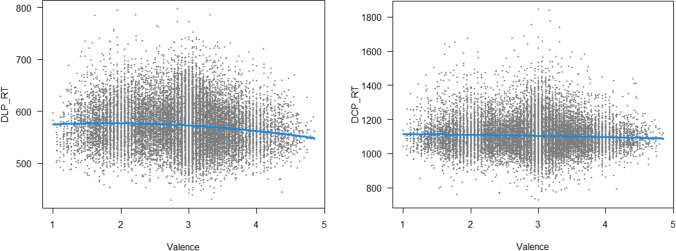
Effect of valence on reaction time (in ms) in the Dutch Lexicon Project 2 (*left side*) and the Dutch Crowdsourcing Project *(right side*), showing that lexical decisions are faster to words with a positive connotation

We started with valence. Each regression used a baseline model of lexical variables taken from Brysbaert et al. (2016): word frequency, word prevalence, number of letters, number of syllables, OLD20 (similarity to other words), and age of acquisition. The model accounted for 48.4% of variance in DLP2 and 65.2% of variance in DCP. Adding a second order polynomial of valence, increased the percentage of variance explained with .6% in DLP2 and .1% in CDP. Both additions were small but significant (*p* < .001) and they agree with the pattern revealed by Gao et al. (2022) for visual lexical decision: Almost equal times for negative and neutral words, and faster responses to positive words (Fig. 8).

Ideally, we would have a database of auditory lexical decisions times, to test the difference for negative words between visual and auditory lexical decision (Gao et al., 2022). Although such a database exists (Ernestus & Cutler, 2015), it is rather small (only 1000 overlapping words) and the percentage variance explained by the base model is only 18% with no significant additional effect of valence.

Since positive valence facilitated lexical decision response time, and due to the large number of words having happiness as the dominant emotion, we wanted to check whether any of the discrete emotions better accounted for differences in lexical decision times than valence. We compared the model including valence with models including each of the discrete emotions in terms of AIC and BIC (with the lower value indicating the best model). This analysis showed that valence was the best predictor of response time in the DLP2 and the DCP, suggesting previously observed effects of valence are not driven by a single emotion. Disgust was the best discrete emotion predictor for both DLP2 and DCP response times (see Table 4).

**Table 4:** AIC and BIC values for models testing effect of valence and single emotions for lexical decision reaction time in the DLP2 and DCP

	DLP2		DCP	
	AIC	BIC	AIC	BIC
Valence	244537.3	244634.4	280755.4	280852.4
Happiness	244697.2	244786.1	280797.8	280886.7
Anger	244757	244845.9	280801	280889.9
Fear	244785.3	244874.3	280833.3	280882.2
Sadness	244800.4	244889.3	280809	280897.9
Disgust	244679.3	244768.3	280793	280881.9
Surprise	244793.8	244882.7	280824.2	280913.1

We also tested whether the discrete emotions explained any additional variance over valence. We compared baseline models including valence with models with each discrete emotion added. Although ratings of happiness, fear, sadness, and disgust explained additional variance in the DLP2 data, and disgust and surprise ratings explained additional variance in the DCP data, the amount of variance explained was extremely low, ranging from .08% to .1% (see Table 5).

**Table 5:** Delta *R*^2^ and *p* values for models testing unique effect of discrete emotions over valence for lexical decision reaction time in the DLP2 and DCP

	DLP2		DCP	
	Δ*R*^2^	*p*	Δ*R*^2^	*p*
Happiness	.0004	< .001	0	.411
Anger	0	.331	0	.873
Fear	.0003	< .001	0	.088
Sadness	.001	< .001	0	.458
Disgust	.0008	< .001	0	.040
Surprise	0	.221	.0002	< .001

### Personality traits

**Fig. 9:**
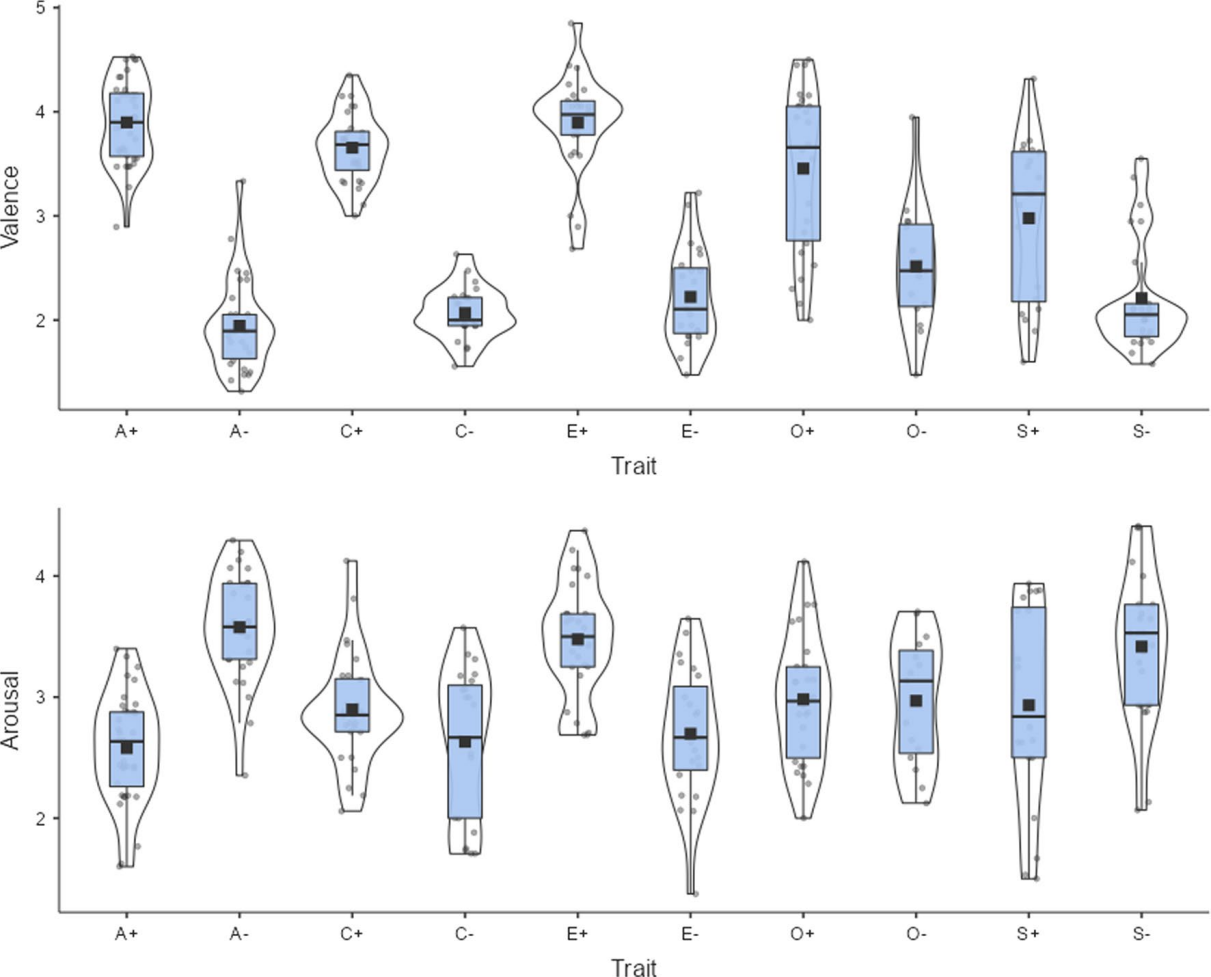
Valence and arousal associated with Big Five personality traits in Dutch. A+/A- = positive/negative pole of agreeableness, C+/C- = positive/negative pole of conscientiousness, E+/E- = positive/negative pole of extraversion, O+/O- = positive/negative pole of openness, S+/S- = positive/negative pole of emotional stability

In Figs. 1 and 2, we saw how English valence and arousal ratings can be used to investigate attitudes to personality traits. Here we tested whether the Dutch emotion norms can also be used in this way. We translated the Goldberg (1992) adjectives into Dutch. In addition, we used the list of Dutch adjectives published by de Raad (2006) to capture the poles of the Big Five traits, together with the nouns and verbs published by de Raad (1992). The data was analyzed in jamovi (The jamovi project, 2022). In total, we had all information for 252 relevant words (about 25 per pole). Example words high on agreeableness were *barmhartig* (merciful), *goedaardig* (benign), *welwillend* (benevolent); words low on agreeableness were: *afkraken* (knock off), *autoritair* (authoritarian), *egoïstisch* (selfish). Words high on conscientiousness were: *doorzetter* (go-getter), *efficiënt* (efficient), *zorgvuldig* (diligent); words low were: *achteloos* (careless), *gemakzuchtig* (easygoing), *nalatig* (negligent). Words high on extraversion were: *actief* (active), *energiek* (energetic), *feestnummer* (party number); words low were: *bedeesd* (timid), *gesloten* (closed), *teruggetrokken* (withdrawn). Words high on openness were: *artistiek* (artistic), *creatief* (creative), *intelligent* (intelligent); words low were: *behoudend* (conservative), *conformist* (conformist), *oppervlakkig* (shallow). Words high on emotional stability were: *aandurven* (to dare), *beslissen* (to decide), *koelbloedig* (cold-blooded); words low were: *bang* (afraid), *dubben* (to doubt), *huilerig* (tearful). Figure 9 shows the distribution of the valence and arousal values for the Dutch words. They largely replicate the findings in English (Figs. 1 and 2), except for one difference: In the Dutch ratings, traits related to introversion are judged more negatively than traits related to extraversion. Dutch-speaking people also seem to experience more arousal in traits related to emotional stability.

**Fig. 10:**
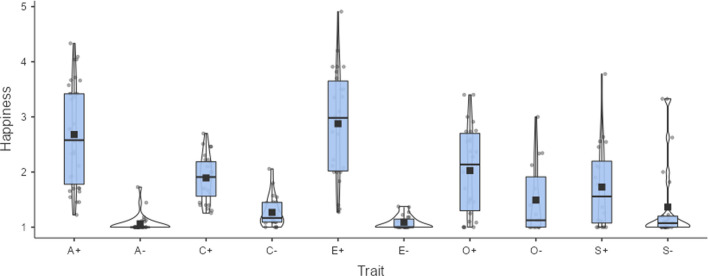
Experiences of happiness associated with Big Five personality traits. A+/A- = positive/negative pole of agreeableness, C+/C- = positive/negative pole of conscientiousness, E+/E- = positive/negative pole of extraversion, O+/O- = positive/negative pole of openness, S+/S- = positive/negative pole of emotional stability

The availability of discrete emotions allow us to estimate the emotions underlying personality traits. As expected, the happiness ratings closely follow the valence ratings (Fig. 10). However, the absolute values of happiness are much lower than the ratings of valence, suggesting that other factors are involved as well. Research by Britz et al. (2022) suggests that social desirability may be one of these factors. Indeed, the authors reported a correlation of *r* = .99 between ratings of valence and social desirability for 500 trait adjectives. So, the high valence ratings for traits of agreeable people is not in the first place because such traits make us happy, but because their behavior is socially desirable. At the same time, there seems to be very little in traits associated with non-agreeable persons, non-conscientious people, introvert persons and emotionally unstable people that makes the participants we tested happy.

**Fig. 11:**
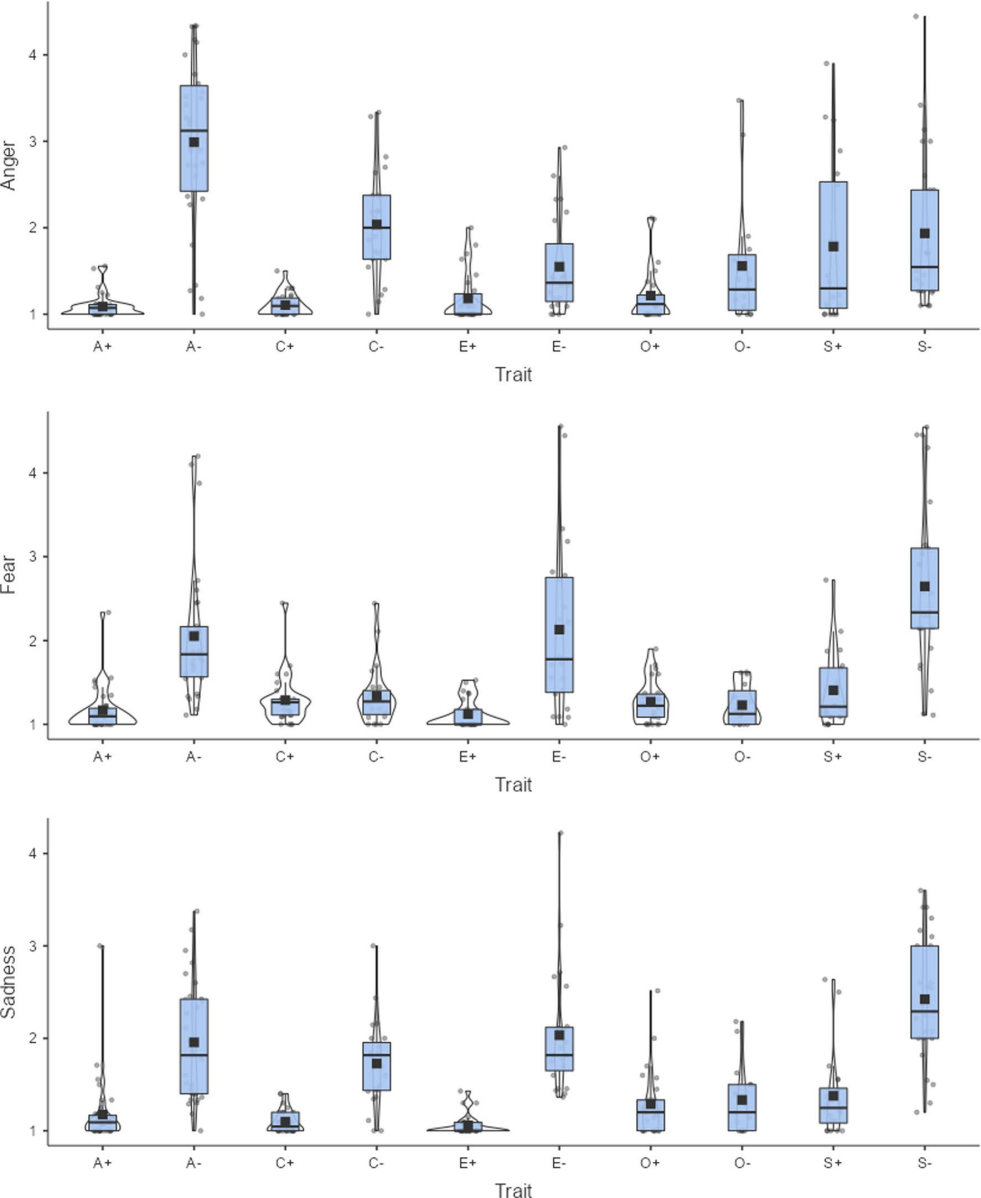

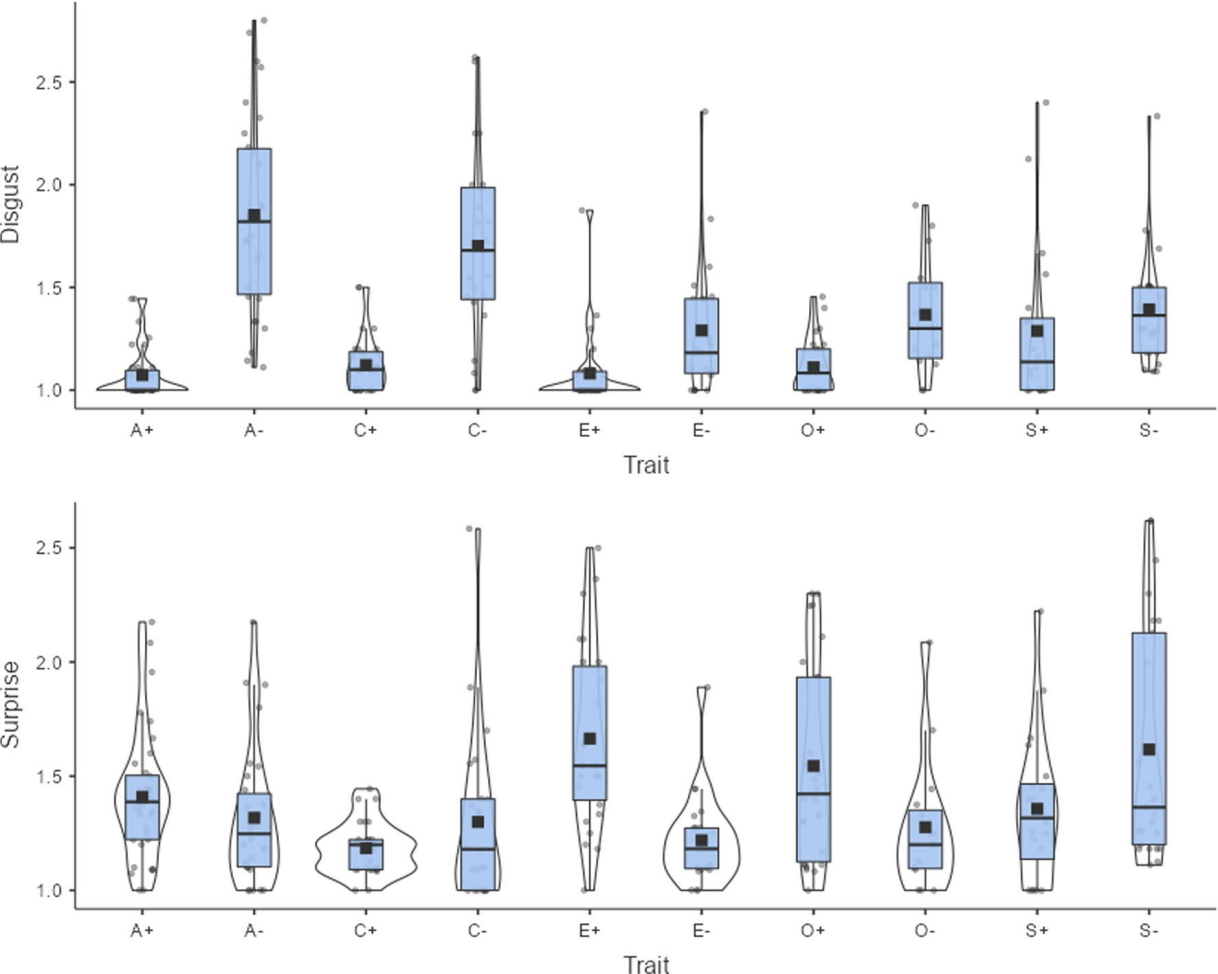
Anger, fear, sadness, disgust, and surprise associated with Big Five personality traits. A+/A- = positive/negative pole of agreeableness, C+/C- = positive/negative pole of conscientiousness, E+/E- = positive/negative pole of extraversion, O+/O- = positive/negative pole of openness, S+/S- = positive/negative pole of emotional stability

Figure 11 shows the associations of other discrete emotions with the Big Five personality traits. Anger is particularly elicited by traits associated with non-agreeable people. Anxiety and sadness are elicited mainly by traits associated with emotionally unstable people and, surprisingly, also to some extent by traits associated with introverts. Traits associated with non-agreeable and unconscientious people also appear to elicit some feelings of disgust. Nothing about personality traits seems to have surprised our participants. Overall, our data show how the new Dutch ratings can be used to explore words specifically related to personality dimensions, revealing emotions that are associated with specific personality traits. Thus, our ratings can be a useful resource for researchers beyond those interested in word processing and word meaning.

## Discussion

We provide a new set of ratings for a large number of Dutch words for emotional valence, arousal, and discrete emotion categories. The new ratings build on previous sets of ratings by including a considerably larger number of words across word classes. The new set of ratings serves as an invaluable resource for continuing research into emotion, such as for conducting well-controlled experiments into the role of emotion in language processing and examining patterns of emotion in text corpora.

We tested the role of emotional valence on Dutch lexical decision responses for the first time, finding a comparable pattern to that found for English (Gao et al., 2022): responses to positive words are faster than negative and neutral words. This contradicts previous suggestions of a symmetrical effect of positive and negative emotion (Kousta et al., 2009; Kuperman et al., 2014; Vinson et al., 2014; Yap & Seow, 2014). Positive words may act as reinforcers (Gao et al., 2022), with the associated positive information facilitating the processing of the word. Future work should ascertain whether this effect differs in the auditory modality, as seen for English, which would support an effect of emotion on sensory-perceptual components of word processing (Gao et al., 2022).

A positivity-bias for the Dutch language was visible in other ways too, supporting a Pollyanna effect observed across languages (Dodds et al., 2015; Garcia et al., 2012). Although the mean valence rating across words was around the middle of the scale, more Dutch words had positively valenced ratings. In addition, valence was positively correlated with word frequency and prevalence, and negatively with word length. Ratings of the discrete emotion categories also showed that the largest proportion of words is associated with happiness.

Personality characteristics were shown to be differentially related to valence and arousal and the discrete emotion categories. As expected, traits associated with agreeable, conscientious, extroverted, open, and emotionally stable people are perceived as more positive, while traits associated with extraverts, unagreeable, and emotionally unstable people were rated as more arousing. This largely follows the patterns observed in English (Goldberg, 1992; Hollis et al., 2017). Our ratings also allowed us to assess how personality characteristics are associated with discrete emotion. We see for example that traits related to people high on agreeableness are more associated with happiness, while traits associated with people low on agreeableness are more associated with anger, fear, sadness, and disgust.

The new ratings are invaluable to ongoing research into language processing. Emotional processing is considered fundamental in many theories of language comprehension. For example, modern hybrid theories of semantics (e.g., Borghi et al., 2019; Connell, 2019) consider sensorimotor simulation, linguistic distributional processing, and emotion simulation as critical processes underlying language comprehension. Yet while many experimental studies and megastudies have provided evidence for the role of sensorimotor simulation and linguistic distributional processing in a variety of lexical tasks, few have taken into account words’ emotional associations. The present set of ratings can be used as additional predictors of lexical processing in existing megastudy data, or used to carefully design stimuli to experimentally tease apart the role of emotion processes in language comprehension. It has been suggested, for example, that words related to olfaction do not engage mental simulation of odor, but may instead be more strongly grounded in emotion (Speed & Majid, 2020). This is supported by the finding that odor- and taste-related words tend to be found in more emotional contexts in language than other sensory-related words (Winter, 2016). If emotional associations play a critical role in language comprehension, further research could explore how this may be affected by context and individual differences, and ultimately how these associations may affect behavior.

Emotional ratings are also useful in understanding how people communicate emotion. Using ratings of happiness and corpora from ten languages across a range of genres, Dodds et al. (2015) observed what they called a “universal positivity bias”: the median perceived average happiness rating of the 5000 most frequently used words exceeded the neutral midpoint across all languages and corpora used. This supports the human tendency to be positive and socially motivated. Beyond fundamental research, the ratings are also of value in applied settings, such as in communication in healthcare (Stortenbeker et al., 2018) or in the use of chatbots (Yun & Park, 2022).

Another important use for emotion ratings is in the analysis of large sets of real-world linguistic data, such as that found on the Internet. For example, sentiment analysis uses word dictionaries with emotion ratings to calculate an overall sentiment for given topics. By including over 24,000 words, our dataset allows a better estimate of emotion in large Dutch text corpora than ever before. Quantifying the emotion of social media messages, for example, could help understand online behavior and the spread of information, including misinformation and fake news. It has been shown, for example, that engagement in online articles about climate change is predicted by emotion words in the article’s headline (Xu et al., 2022).

It should be noted that while we consider valence and arousal as separable dimensions here, research suggests that the two dimensions may not be so easily separated (Kron et al., 2013, 2015). When emotional valence of images is measured with separate unipolar scales (one for pleasant and one for unpleasant), valence measures largely overlap with measures of arousal (Kron et al., 2013, 2015). One reason suggested for this is that a bipolar scale is unable to correctly measure the valence associated with concepts of mixed valence, for example, “bitter-sweet” feelings, also referred to as valence ambiguity (Mattek et al., 2017). Such concepts would typically be rated close to neutral on a bipolar scale, meaning that the valence associated with the concepts is compressed, while actually being strongly correlated with unipolar valence and arousal. Future research should explore the role of valence and arousal in word meaning and word processing using different measures of valence, including unipolar and bipolar measures, as well as measures of valence ambiguity, to explore both the role of measurement scale used as well as to assess how models of emotion can be applied to ambiguous concepts.

## Conclusion

Our new set of emotion norms demonstrates the key role emotion plays in word meaning. We broadly replicate findings previously demonstrated with English, including the relationship between emotion and key lexical variables as well as personality characteristics. Our emotion norms are a useful resource for ongoing research in language processing as well as work in applied settings where emotional communication is crucial.

## Data Availability

The data, stimuli, and analysis files are available via the Open Science Framework (https://osf.io/9htuv/).

## Footnotes

1 An exception was made for the valence ratings, where an equal number of men and women were tested to look for gender differences in the ratings. Unfortunately, the information about the gender of the participants was lost when the data were anonymized.
